# Metabolic Control of Autoimmunity and Tissue Inflammation in Rheumatoid Arthritis

**DOI:** 10.3389/fimmu.2021.652771

**Published:** 2021-04-02

**Authors:** Jingtao Qiu, Bowen Wu, Stuart B. Goodman, Gerald J. Berry, Jorg J. Goronzy, Cornelia M. Weyand

**Affiliations:** ^1^ Department of Medicine, Stanford University School of Medicine, Stanford, CA, United States; ^2^ Department of Orthopedic Surgery, Stanford University School of Medicine, Stanford, CA, United States; ^3^ Department of Pathology, Stanford University School of Medicine, Stanford, CA, United States

**Keywords:** T cell, metabolism, autoimmunity, rheumatoid arthritis, mitochondria, glycolysis, glutaminolysis, fatty acid

## Abstract

Like other autoimmune diseases, rheumatoid arthritis (RA) develops in distinct stages, with each phase of disease linked to immune cell dysfunction. HLA class II genes confer the strongest genetic risk to develop RA. They encode for molecules essential in the activation and differentiation of T cells, placing T cells upstream in the immunopathology. In Phase 1 of the RA disease process, T cells lose a fundamental function, their ability to be self-tolerant, and provide help for autoantibody-producing B cells. Phase 2 begins many years later, when mis-differentiated T cells gain tissue-invasive effector functions, enter the joint, promote non-resolving inflammation, and give rise to clinically relevant arthritis. In Phase 3 of the RA disease process, abnormal innate immune functions are added to adaptive autoimmunity, converting synovial inflammation into a tissue-destructive process that erodes cartilage and bone. Emerging data have implicated metabolic mis-regulation as a fundamental pathogenic pathway in all phases of RA. Early in their life cycle, RA T cells fail to repair mitochondrial DNA, resulting in a malfunctioning metabolic machinery. Mitochondrial insufficiency is aggravated by the mis-trafficking of the energy sensor AMPK away from the lysosomal surface. The metabolic signature of RA T cells is characterized by the shunting of glucose toward the pentose phosphate pathway and toward biosynthetic activity. During the intermediate and terminal phase of RA-imposed tissue inflammation, tissue-residing macrophages, T cells, B cells and stromal cells are chronically activated and under high metabolic stress, creating a microenvironment poor in oxygen and glucose, but rich in metabolic intermediates, such as lactate. By sensing tissue lactate, synovial T cells lose their mobility and are trapped in the tissue niche. The linkage of defective DNA repair, misbalanced metabolic pathways, autoimmunity, and tissue inflammation in RA encourages metabolic interference as a novel treatment strategy during both the early stages of tolerance breakdown and the late stages of tissue inflammation. Defining and targeting metabolic abnormalities provides a new paradigm to treat, or even prevent, the cellular defects underlying autoimmune disease.

## Introduction

Rheumatoid arthritis (RA) is a chronic, systemic autoimmune disease characterized by inflammation of the synovial tissue and autoantibody production (1, 2). Typically, the synovial membrane is infiltrated with T cells, B cells and macrophages, eliciting a maladaptive response-to-injury in fibroblast-like synoviocytes (FLS) that hyper-proliferate and destroy cartilage and bone (1, 3). Over the decade-long course of RA, a combination of genetic, epigenetic, and environmental factors contributes to rendering the host susceptible to autoimmunity and the eventual appearance of joint inflammation. An array of immune cells, including macrophages, dendritic cells, mast cells, neutrophils, T cells and B cells, have all been implicated in the disease process and make a pathogenic contribution during early loss of self-tolerance, the subsequent progression to joint inflammation when joint-specific protection factors fail and the final “non-healing wound” when the collective action of immune cells and stromal cells demolishes the joint (1, 4).

Patients diagnosed with RA produce a specific set of autoantibodies, typically reactive against post-translational modifications of proteins (5–7). Such autoantibodies appear decades before clinically apparent disease (8). Reactivity against a broad spectrum of citrullinated proteins, instead of a single autoantigen, has questioned the relevance of classical models of autoimmunity in RA and instead has emphasized the potential role of antigen-nonspecific factors, such as the metabolic control of immune cell function (9, 10). The genetic risk conferred by HLA class II molecules, the accumulation of chronically activated T cells in the diseased joint and the T-cell dependence of autoantibody production all support a critical role of dysfunctional memory T cells as a salient feature of RA. Differentiation and functional commitment of T cells is critically dependent on metabolic adaptations that co-ordinate the biosynthetic and energy demands imposed by chronic activation and the massive generation of cellular offspring (11). It is now clear that T cells not only differentiate into memory T cells, they also can become aberrantly activated, phenotypically unstable, exhausted, or senescent (12, 13). All these different functional states are ultimately interlinked with a specific metabolic program. RA T cells are characterized by a metabolic signature and progress has been made in defining molecular mechanisms underlying the metabolic deviations. Here, we will review how T cell metabolism influences the development of inflammation in the synovial tissue, focusing on three major metabolic pathways: glucose metabolism, glutamine metabolism and lipid metabolism. Ultimately, understanding how energy utilization and metabolite production dictates the cells’ fate outside and inside of the joint will set the stage for the design of novel immunotherapeutic strategies.

## Glucose Metabolism as an Arthritogenic Risk Factor

Glucose metabolism, one of the primary metabolic pathways in the body, primarily refers to the process of breaking down glucose into ATP and intermediate metabolites, including glycolysis, aerobic oxidation, and processing in the pentose phosphate pathway (PPP). Glucose metabolism not only provides energy for physical activity but also mediates a variety of physiological processes through the formation of complex signaling networks with metabolic substrates. Glycolysis involves the catalytic conversion of one molecule of glucose into lactic acid, producing two adenosine triphosphate (ATP) molecules, whereas aerobic oxidation catalyzes one molecule of glucose into CO_2_ and H_2_O and generates 38 or 36 ATP molecules. Both glycolysis and aerobic oxidation are important ways to generate ATP, but the choice is typically adaptive to the physiological and pathological needs occurring in cells, tissues, and organs. In recent years, data have accumulated identifying glucose metabolism as a key component in the pathogenesis of RA.

### Glycolytic Breakdown in Inflammatory Effector Cells

**Figure 1:**
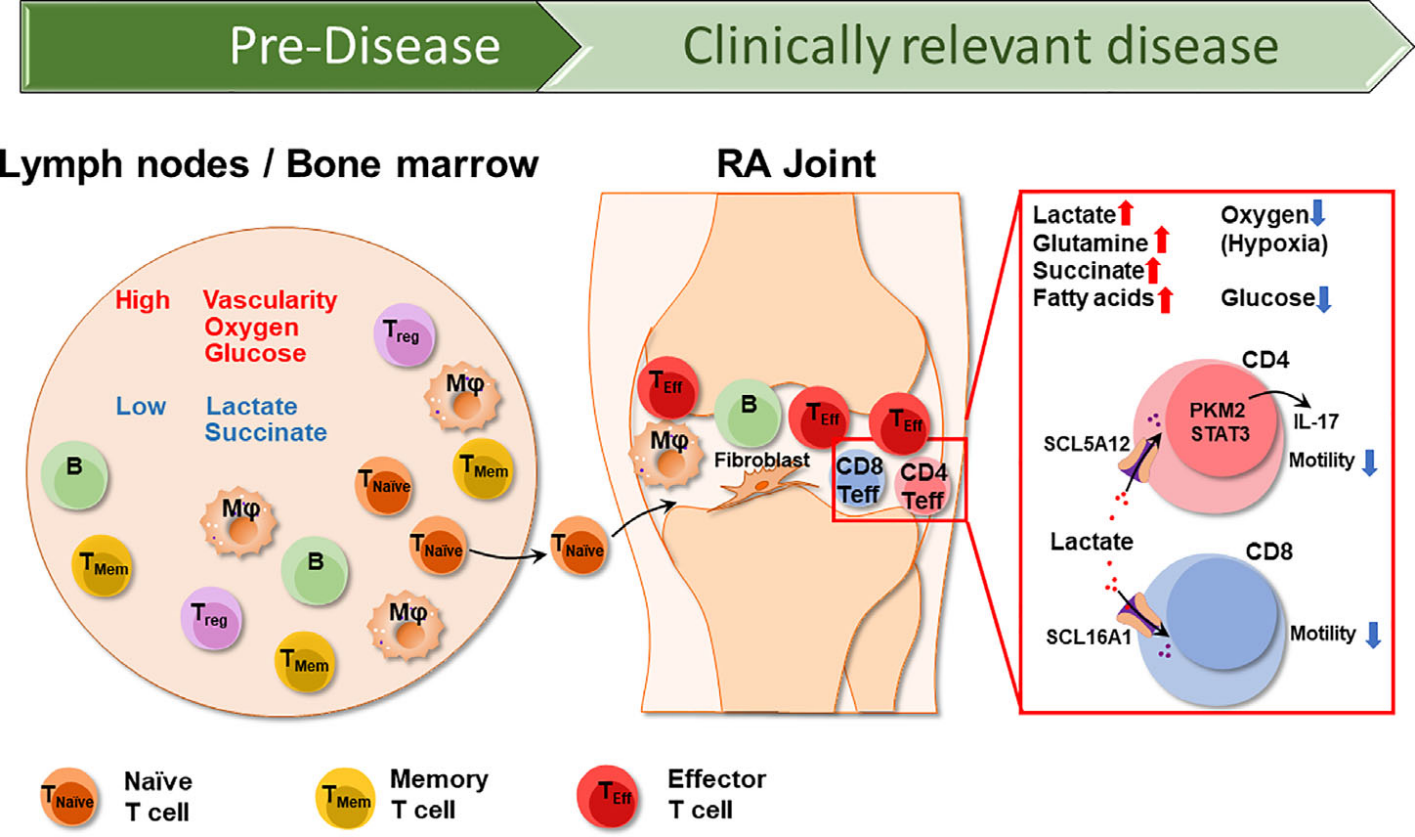
The different Tissue Environments of Rheumatoid Arthritis. Early events in the RA disease process, e.g., the loss of self-tolerance of RA T cells occurs in lymphoid tissues, such as lymph nodes and the bone marrow. Over lifetime, lymphoid tissues remain the home of naïve and memory T cells, which circulate through the peripheral blood where they can be sampled. Lymph nodes are highly vascularized, therefore have access to oxygen and glucose. As RA naïve T cells differentiate into short lived effector T cells (SLEC), they acquire the ability to leave the circulation and invade into the synovial tissue. Synovial effector T cells encounter a different metabolic environment which is hypoxic, low in glucose and high in lactate due to the chronic activation of macrophages and stromal cells. CD4^+^ effector T cells and CD8^+^ effector T cells uptake lactate through SCL5A12 and SCL16A1, respectively. Imported lactate promotes IL-17 secretion in CD4^+^ T cells and inhibits both CD4^+^ and CD8^+^ T cell motility, essentially arresting T cells in the lactate^hi^ synovium. T _naïve_, naïve T cell; T_Mem_, memory T cell; T_reg_, regulatory T cell; Mφ, macrophage; B, B cell.

In normal synovial tissues, glycolysis is the primary pathway promoting mitochondrial substrate oxidation of pyruvate. The activity levels of glyceraldehyde 3-phosphate dehydrogenase (GAPDH) and lactate dehydrogenase (LDH), the major enzymes of the glycolytic pathway, are increased in RA synovial cells (14). Accordingly, lactate concentrations are high and glucose concentrations are distinctly low in the inflamed joint (15). Hexokinase, which catalyzes the first step in glucose metabolism, enhances the ability of FLS to migrate and invade (16, 17). In RA FLS, the balance between glycolysis and oxidative phosphorylation is shifted toward glycolysis (18). Synovial fibroblasts are key effector cells in the final stages of RA (19), are under considerable metabolic stress and produce competition for energy sources. Synovial tissue with its increased glycolytic activity represents only one “battle ground” of the disease. Disease-relevant cells live and function in other tissue environments, particularly the lymphoid tissues from which they originate (**Figure 1**).

Accordingly, naïve CD4^+^ T cells isolated from RA patient exhibit diminished glycolytic activity (20). Before such naïve CD4^+^ T cells become pathogenic memory and effector T cells, they utilize glucose in a distinctly different manner than naïve CD4^+^ T cells from healthy individuals: they avoid glycolytic breakdown into lactate and instead divert glucose into the PPP, driving the accumulation of NADPH and consumption of cellular reactive oxygen species (ROS). With an excess of reducing equivalents, T cells are unable to activate the relevant redox kinases, which allows them to bypass the regulatory checkpoints of the G2/M cell cycle and enter unrestrained proliferation (21). The lack of cellular ROS appears to be a T-cell-specific feature.

**Figure 2:**
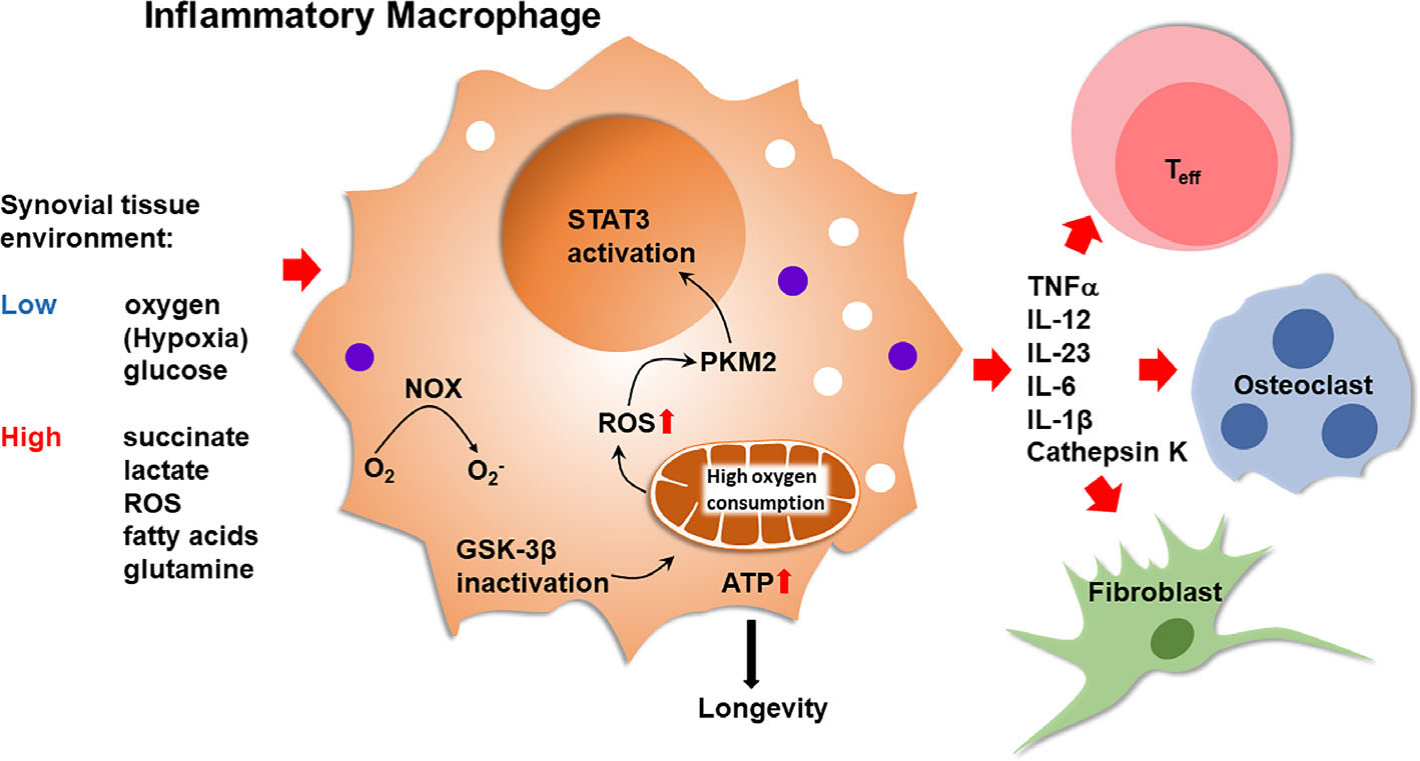
Inflammatory Macrophages in Rheumatoid Synovitis. Bone marrow-derived macrophages that infiltrate into the arthritic joint enter an oxygen^lo^, glucose^lo^, ROS^hi^, succinate^hi^, fatty acid^hi^, lactate^hi^ tissue environment. Inactivation of glycogen synthase kinase 3β (GSK-3β) in macrophages optimizes pyruvate import, enhancing mitochondrial activity. High oxidative phosphorylation increases ATP production and ROS release. ATP abundance promotes macrophage longevity. ROS facilitate the dimerization of the cytosolic enzyme pyruvate kinase M2 (PKM2), enabling nuclear translocation and STAT3 activation by the kinase. High mitochondrial activity supports the production of TNFα, IL-12, IL-23, IL-6 and IL-1β. Inflammatory macrophages trigger and sustain synovitis by modulating the function of neighboring T effector cells, endothelial cells, osteoclasts and synovial fibroblasts. NOX, NADPH oxidase; T_eff_, T effector cell.

Macrophages from RA patients have high mitochondrial activity and can efficiently generate ROS (22–24). In patient-derived macrophages, suppression of GSK-3β fuels mitochondrial activity, enhances ATP synthesis and ROS release. This metabolic constellation causes the cytoplasm-to-nucleus translocation of the glycolytic enzyme pyruvate kinase M2 (PKM2). Functional outcomes include the PKM2-dependent activation of STAT3, boosting the production and secretion of pro-inflammatory cytokines (25), such as IL-6 and IL-1 (**Figure 2**). Such pro-inflammatory macrophages accompany T cells in the inflamed joint, where both cell types compete for the access to glucose. Naïve CD4^+^ T cells live in lymphoid organs and it is unlikely that inflammatory macrophages regulate the fate decisions of such T cells prior to their differentiation into memory/effector cells. In the inflamed joint, multiple, functionally diverse macrophage subsets are now recognized (26). In general, inflammatory macrophages are considered to mainly rely on glucose as an energy carrier and resolving/anti-inflammatory macrophages are known to require less glucose and supply their bioenergetic and biosynthetic needs through mitochondrial oxidative phosphorylation (27). Whether synovial macrophage subsets differ in their energy generation and utilization and the metabolic environment they build in their surroundings remains unexplored.

### Oxidative Phosphorylation in Inflammatory Effector Cells

**Figure 3:**
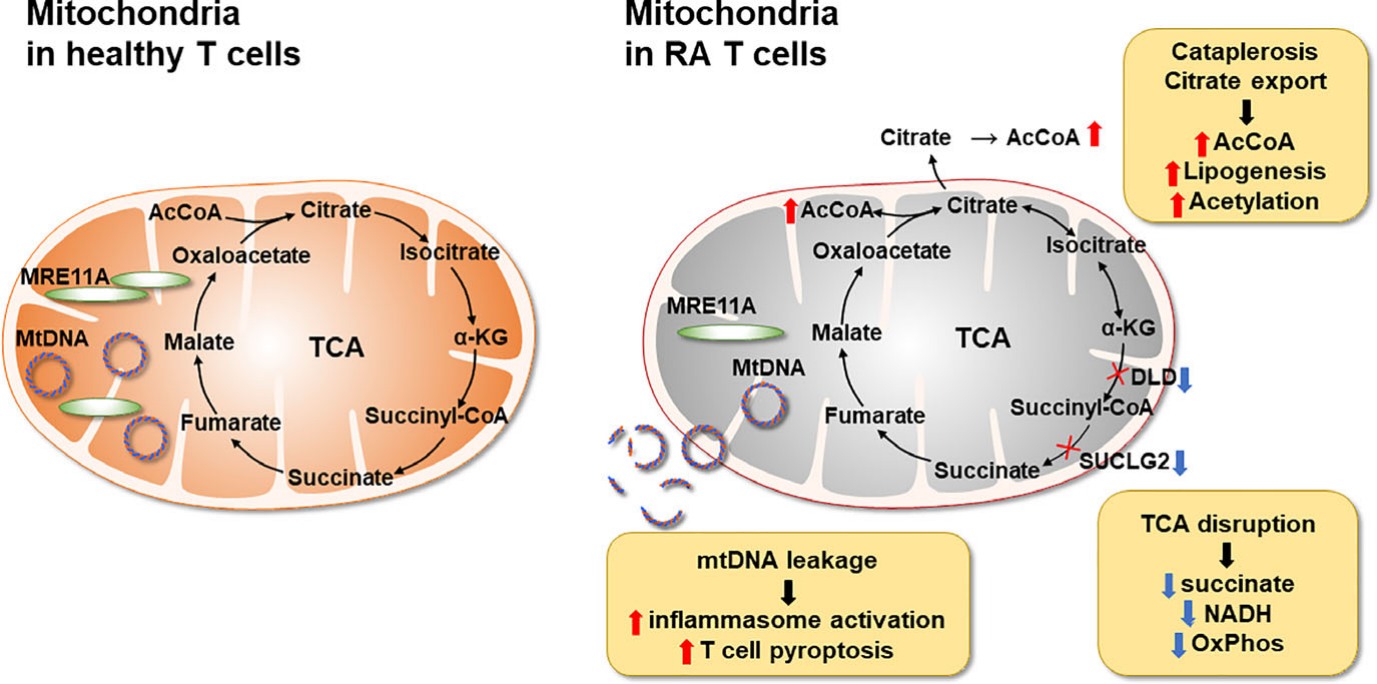
Mitochondrial Defects in T cells in rheumatoid arthritis. RA CD4^+^ T cells fail to maintain mitochondrial DNA due to the transcriptional repression of the nuclease MRE11A, a limiting factor in DNA double-strand break repair and replication fork protection. Instability of mtDNA causes leakage of mtDNA fragments into the cytosol, triggering activation of the inflammasome and eventually T cell pyroptotic death. Mitochondrial oxidative phosphorylation is further impaired by the disruption of the TCA cycle. Transcriptional repression of succinyl-CoA ligase (SUCLG2) prevents the conversion of a-ketoglutarate into succinate and forces the TCA cycle to change direction. In a cataploretic reaction, mitochondria of RA T cells produce and export excess citrate, creating an acetyl-CoA-rich environment in the cytoplasm. Together with NADPH generated in the hyperactive pentose phosphate pathway surplus acetyl-CoA enters biosynthesis, specifically lipogenesis. Also, excess acetyl-CoA drives protein acetylation, including the acetylation of microtubules. α-KG, α-ketoglutarate; mtDNA, mitochondrial DNA; SUCLG2, Succinate-CoA Ligase GDP-Forming Subunit Beta.

The major metabolic abnormality of RA CD4^+^ T cells lies in the mitochondria (**Figure 3**). Mitochondrial failure presents as low ATP generation and reduced release of ROS (28). Production of the mitochondrial intermediate succinate is impaired, and the mitochondrial tricarboxylic acid cycle (TCA) cycle changes direction (29). Unable to convert acetyl-CoA into ATP, RA CD4^+^ T cells produce excess citrate and transport it out of the mitochondria into the cytosol (29). Here, acetyl-CoA participates in post-translational modification of proteins, shifting the functional commitment of the cells toward mobility and cytokine release (29). Mitochondrial failure in RA T cells results from insufficient repair of mitochondrial DNA, due to loss-of-function of the DNA repair nuclease MRE11A (30). A second dimension in maintaining mitochondrial fitness derives from the misplacement of the energy sensor AMP-activated protein kinase (AMPK) (31). Under physiologic conditions, a decline in ATP would trigger mitochondrial biogenesis *via* activation of AMPK (32). In RA T cells, this mechanism is paralyzed, forcing the cell to function with damaged mitochondria. The ATP^lo^ status of RA T cells is aggravated by suppressed glycolysis; attributable to the transcriptional repression and functional impairment of the glycolytic enzyme phosphofructokinase (20). Instead, glucose is shunted into the PPP, supporting anabolic metabolism and laying the groundwork for biosynthesis, biomass production and the generation of new cells (21). Together, these data support the concept that irregularities in glucose metabolism are evident during an early stage of RA, when naïve CD4^+^ T cells deviate from a normal differentiation pattern and make a commitment to become a pro-inflammatory effector cell.

### Immunoregulation Through Glucose Uptake

As part of their activation program, T cells switch to high glycolytic flux (33, 34), securing rapid access to ATP, but also to biosynthetic precursor molecules required for cellular expansion. The swift upregulation of glycolysis is linked to the increased expression of the glucose transporter 1 (GLUT1) maximizing glucose uptake by the cell (35). Glucose uptake in T cells undergoing activation is strictly correlated to GLUT1, identifying the transporter as a key determinant in glucose utilization (36, 37). Notably, GLUT1 expression differs among different T cell lineages and is highest in Th17, Th2, and follicular helper T cells (TFH) cells, followed by Th1 cells (38, 39). This difference in expression correlates with differential glycolytic rates in these T cell subsets (38) and identifies GLUT1 as a potential target for immunomodulation. Induction of *GLUT1* transcripts is an early event during T cell receptor stimulation and is well known to depend on co-stimulatory signals. Signaling through CD28 and the downstream PI3K/Akt activation determines glucose uptake though GLUT1 upregulation (40). Cross-linking of the T cell receptor alone or in combination with IL-2 appears to have no effect on the level of GLUT1 expression (41, 42). Like T cells, B cells require GLUT1 upregulation for optimal activation responses and similarly, rely on PI3K-dependent signaling (43).

Experimental data suggest that GLUT1-mediated glucose transport may have differential effects in CD4^+^ and CD8^+^ T cells. GLUT1 knockout (KO) T cells fail to proliferate in response to immunization with an experimental antigen or *in vitro* stimulation (44). Similarly, treatment with CG-5, a nonselective small molecule glucose transport inhibitor inhibited T cell proliferation (45). CG-5 blocked Th1 and Th17 polarization and reduced T cell expansion in a mixed lymphocyte reaction. Interestingly, GLUT1^neg^ CD8^+^ T cells proliferated to a similar extent and expressed equal levels of granzyme B as their wild type counterparts following stimulation. Thus, the cytotoxic potential of CD8^+^ T cells may not depend on GLUT1. Increased expression of GLUT3 and GLUT6 in GLUT1 KO CD8^+^ T cells could possibly compensate for the deficiency of glucose uptake by GLUT1 (44). More importantly, immunosuppressive T regulatory cells were not affected by GLUT1 deletion as they rely on fatty acid oxidation rather than glycolysis for their activity (38). GLUT1 expression had major consequences not only for proliferation but also for differentiation of T cells, as exemplified by the deficient induction of IFN-γ and IL-17 in GLUT1^lo^ T cells (45).

The function of GLUT family member in T cells is well studied, but not clear in RA. There are only a few reports on GLUT1, GLUT3 and GLUT4 in RA. Heterozygous deletion of *GLUT3* correlates directly with expression levels of GLUT3 and influences glycolysis rates in the human immune system (46), but the frequency of the GLUT3 copy number variant is not different among RA, multiple sclerosis and heathy control, providing no evidence for RA protection in deletion of GLUT3 (46), the study of which demonstrated GLUT3 is not necessary for glucose transfer in patients with RA. However, another study showed the mutual activation between CD4^+^ T cells and FLS, which resulted in increased proliferation and expression of glucose transporters GLUT1 and GLUT3 in FLS (47). In rat Arthritis model, arthritic rats showed cachexia, reduced adipocyte size, and downregulated GLUT4 in adipocyte membranes (48). Although the function of GLUT4 in pro-inflammatory T cells is unknown, the downregulated membrane GLUT4 confirms its role in autoimmunity.

Taken together, the intensity of glycolysis is highly dependent on active glucose transport, making GLUT1 an excellent marker of glycolytically active T cell populations. Glucose utilization determines T cell proliferation and differentiation, identifying this metabolite as an excellent candidate for metabolic interference. Immunoinhibitory Treg cells are less dependent on glucose uptake and should therefore be less vulnerable to glucose withdrawal. Controlling glucose import may represent an excellent approach to immunomodulation.

### Tissue Lactate as an Immunoregulatory Mediator

Glycolysis has a low efficiency in generating energy. The terminal product of the anaerobic oxidation of glucose is lactate. Lactate has long been considered a “waste” by-product of cell metabolism, and it accumulates at sites of high glycolytic activity, such as tumor surroundings or inflamed tissue areas. Clinically, elevated serum lactate levels are a sensitive indicator of stress-induced glycolysis during sepsis (49). Since lactate production acidifies the environment, physiologic lactate concentrations are kept in a narrow range (1.5-3 mM) in blood and tissues of healthy individuals. Lactate can serve as an energy carrier and can be transported across cell membranes by mono-carboxylate transporters and can be reabsorbed by the kidneys to prevent energy loss. However, fueled by extensive glycolysis, lactate can increase up to 10 mM in inflammatory lesions and even higher (30-40 mM) in cancerous tissues (50, 51). Lactate accumulates in the synovial fluid of RA patients (52), reflecting high glucose turnover by cells trapped in the synovial membranes. It has been proposed that synovial lactate measurement may function as a reliable indicator to differentiated subtypes of inflammatory arthritis (53) (**Figure 1**).

Elegant work has examined the immunomodulatory impact of synovial tissue lactate in RA patients. T cells sense lactate *via* the expression of specific transporters and RA T cells may even be more reliant on the uptake of energy carriers, given their inherent difficulties of generating ATP in their mitochondria. Lactate uptake by CD4^+^ T cells has been implicated in inhibiting migratory capability, essentially arresting T cells in the lactate^hi^ microenvironment (**Figure 1**). Also, lactate availability fostered differentiation of a Th17 subset, further supporting the chronic inflammatory process (49, 54). As reported by Pucino et al., synovial T cells adapt to their milieu by upregulating the lactate transporter SLC5A12. In line with the fundamental difficulties of RA T cells to secure energy production through mitochondrial activity (29, 30), SLC5A12 high-expressing tissue CD4^+^ T cells appear to be particularly dependent on utilizing alternative energy resources. Nurtured by metabolically highly active macrophages and synovial cells (18, 55) lactate-depending CD4^+^ T cells will be functionally biased toward IL-17 production, mediated by nuclear PKM2/STAT3 and enhanced fatty acid synthesis (56). Here, tissue-resident T cells display a metabolic signature previously described for pro-inflammatory macrophages that use nuclear PKM2/STAT3 to sustain IL-1 and IL-6 production (57).

Furthermore, tissue lactate not only shapes the functional commitment of CD4^+^ T cells, but also affects CD8^+^ effector T cells. Extracellular sodium lactate and lactic acid inhibit the motility of CD4^+^ and CD8^+^ T cells, respectively. The selective regulation of T cell mobility is mediated *via* subtype-specific transporters, SLC5A12 and SLC16A1, selectively expressed on CD4^+^ and CD8^+^ T cells, respectively (50). Underlying mechanisms have been defined and show that the lactate-induced inhibition of CD4^+^ T cell movement results from an interference with glycolysis that is activated when the chemokine CXCL10 engages the chemokine receptor CXCR3. CXCR3 is typically expressed on effector T cells, both CD4^+^ and CD8^+^ subtypes (58), emphasizing that the lactate-rich environment modifies the function of differentiated effector T cells as opposed to lymph node-residing naïve T cells that are upstream of synovitis induction (**Figure 1**). Interestingly, lactate exposure appears to result in a very different outcome for CD4^+^ and CD8^+^ effector T cells, stimulating the prior and inhibiting the latter (50). Single cell technology may be helpful in further breaking down the heterogeneous nature of tissue-residing effector T cells (59, 60), enabling the assignment of functional subsets to particularly patterns of nutrient uptake and utilization.

Lactate has drawn broad attention as a metabolite shaping the tissue milieu within and around tumors. A positive correlation between lactate dehydrogenase A (LDH-A), high local lactate levels, and tumor progression has been documented in various tumors (61). Autoimmunity-related tissue inflammation causes less of an acidification, but infiltrating cells may be exposed to similar cues. LDH isoenzymes have been reported to be higher in serum and synovial fluid of RA compared to osteoarthritis patients (62). And, it has long been known that LDH activity is increased in RA synovial tissues compared to healthy controls, emphasizing the dependence of the lesion on glycolytic metabolism (63). In healthy T cells, LDH-A is important in regulating differentiation and lineage assignment. By increasing availability of substrate for acetylation and shifting the T cell epigenome, LDH promotes Th1 commitment and IFN-γ production (64). A recent study has identified LDHA high expression as a feature of all CD8^+^ T cell subsets in RA patients (65). Inhibition of LDHA with FX11 (LDHA inhibitor) led to reduction in lipogenesis, migration, and proliferation of CD8^+^ T cells, and lowered CD8^+^ T cell effector functions (65). LDHA inhibition successfully abrogated the ability of RA CD8^+^ T cells to sway healthy B cells toward a pro-inflammatory phenotype. The LDHA^hi^ phenotype of peripheral CD8 T cells was maintained in CD8^+^ T cells from the synovial membrane (65).

In summary, upregulation of glycolysis, and with it the production of lactate, is a marker of cellular activation and growth. The dependence of highly proliferative cells on glucose as an energy carrier leads to localized lactate accumulation and to acidification of the tissue site. During the late stages of the RA disease process, after T cells have arrived in the tissue environment, they are exposed to a lactate-rich milieu. Lactate providing a cellular arrest signal fits well into the concept that T cells build an extra-lymphoid site where they persist, commit to pro-inflammatory effector functions, and promote the building of self-sustained lymphoid architecture (66–68).

### Immunoregulation Through the Mitochondrial Intermediate Succinate

By producing NADH, the tricarboxylic acid (TCA) cycle is a central route for oxidative phosphorylation, fueling complex I of the electron transport chain and eventually ATP generation. The TCA cycle fulfills other bio-energetic, biosynthetic, and redox balance requirements and functions as a metabolic hub. One major TCA function is the production of metabolic intermediates, that can be transported out of the mitochondria and participate in cytosol, nuclear and extracellular processes. An abundant TCA metabolite is succinate, which, like lactate, appears in the extracellular milieu, where it can be taken up by surrounding cells. Succinate is a product of myeloid and lymphoid cells, but the succinate receptor GPR91 is selectively expressed on monocytes/macrophages, granulocytes, and dendritic cells (69–71). GPR91 plays a critical role in the development of immune-mediated arthritis, supports the expansion of the Th17 cell population and acts as an overall amplifier of experimental antigen-induced arthritis. *GPR91-/-* mice show reduced articular hyperalgesia, neutrophil infiltration and inflammatory cytokines in the joint, and reduced frequency of Th17 cells in the draining lymph nodes (71).

Evidence has been provided describing succinate as a signaling molecule in the arthritic joint, with macrophages as the recipients of the stimulatory signals (70) (**Figure 2**). However, the cellular source of the succinate has not been determined. In general, succinate is recognized as a strong pro-inflammatory stimulator (72). LPS stimulation of macrophages results in abundant succinate production, which stabilizes Hypoxia-inducible factor 1α (HIF-1α) and IL-1β an important downstream target (73). In innate immunity, succinate may be one of the major signaling molecules (72), identifying metabolic activity as a key determinant of regulating the intensity of inflammation. Conversely, in the adaptive immune system, lack of succinate appears to be of particularly relevance in breaking tissue tolerance and causing inflammation (**Figure 3**). T cells isolated from RA patients are distinctly low in succinate, due to repression of the *SUCLG2* gene, which brings the TCA to a halt and shifts from the oxidative to the reductive direction. One outcome is the accumulation of the oncometabolite α-ketoglutarate, feeding the production of citrate and acetyl-CoA. Excess acetyl-CoA imposes a pro-inflammatory effector phenotype through acetylation of the microtubular cytoskeleton (**Figure 3**). Succinate^low^ acetylCoA^hi^ T cells rapidly reshape their cellular body, form a rear uropod, become hypermobile and invade into the tissue site (29). In that scenario, succinate supplement could be beneficial, reestablishing directionality of the TCA cycle, supporting the electron transport chain and forcing the T cell into a more tolerant state. Diametrical metabolic states in innate and adaptive immune cells seems to be a consistent finding in RA (55), beyond the role of succinate. Also, ATP and reactive oxygen species are abundant in monocytes/macrophages but scares in T cells. It is currently unknown how hypermetabolic macrophages communicate with hypometabolic T cells and vice versa and what the functional consequences are. But succinate appears to have a context-specific and cell-type specific role extending beyond its function as an energy carrier.

## Hypoxia as an Amplifier of Tissue Inflammation

Mitochondrial metabolism ultimately depends on the availability of oxygen, which functions as the final electron acceptor in the electron transport chain. Oxygen pressures are highest in the lung, considerably lower in the blood and reach hypoxic levels in the tissue. Immune cells and tissue stromal cells need to adapt to hypoxic conditions and hypoxia is now recognized as a critical modulator of RA tissue inflammation (74). In line with the concept that reduced mitochondrial fitness is a risk factor for pro-inflammatory behavior, levels of synovial oxygen have been reported to negatively correlate with disease activity. Hypoxia induces a wide spectrum of alterations in mitochondrial structure, dynamics, and mitochondrial DNA (mtDNA) stability, resulting in impaired mitochondrial respiration, excessive production of reactive oxygen species (ROS), loss of ATP, increased oxidative damage and the accumulation of mtDNA mutations (75). One consequence of hypoxia is the activation of the transcription factor HIF-1α, which in turn promotes a gene program designed to enhance the production of glycolytic energy, including glucose transporters and glycolytic enzymes (76, 77). Hypoxic conditions in the rheumatoid joint should favor the survival of T cells that are less dependent on glycolytic breakdown, e.g., T cells with repression of the glycolytic enzyme PFKFB3 (78). PFKFB3^lo^ RA T cells shift glucose toward the PPP (20) and produce biosynthetic precursors even under low oxygen conditions. Cells less dependent on proliferative expansion, such as synovial fibroblasts and macrophages, switch toward glycolysis as an oxygen-independent way of generating ATP.

By sustaining chronic upregulation of HIF-1α, synovial hypoxia provides a feed-forward mechanism amplifying synovitis. HIF-1α increases the activity of lactate dehydrogenase A (LDHA), which converts pyruvate to lactate. The resultant acidic environment promotes fibroblast and immune cell proliferation and persistence. High lactate and low O_2_ enable the survival of T cells by stabilizing HIF-1α (79, 80). HIF-1α has been reported to be highly expressed in RA synovium (81), identifying this transcription factor as a hallmark of synovial tissue inflammation. Hypoxia further enhances the stabilization of HIF-1α induced by T-cell-receptor-mediated activation of the PI3K-mTOR pathway (82), functioning as a stabilizer of autoimmune tissue inflammation. HIF-1α^+^ T cells may be particularly adapted to serve as pro-inflammatory effector cells (74). Driven by the hypoxic microenvironment of the joint, FOXP3^+^ regulatory T cells promptly convert to pathogenic Th17 cells (83), further weakening anti-inflammatory mechanisms. HIF-1α-dependent induction of retinoic acid-receptor-related orphan receptor-γt (RORγt) combined with targeting FOXP3 for degradation is detrimental to Treg cells and fosters Th17 cell generation (84), identifying hypoxia as a potent risk factor for unrelenting inflammation.

Low availability of oxygen equally encourages pathogenic traits of stromal cells. Hypoxia and IL-17 synergize to drive migration and invasion of synovial fibroblasts through MMP2 and MMP9 expression (85). HIF-1α also controls fibroblast IL-33 production, which in turn enhances HIF-1α expression and generates a regulatory cycle that perpetuates RA inflammation (86). Also, cooperation of HIF-1α with intracellular signaling cascades may accelerate pro-inflammatory pathways. Under hypoxic conditions, HIF-1α interacts with Notch-3 and STAT-1 in RA synoviocytes to stabilize and enhance stromal cell inflammation (87).

## Glutamine as a Fuel Source for Joint Inflammation

While much of the focus of immunometabolism has been directed to central carbon metabolism, e.g., glycolysis and the TCA cycle, amino acids are now emerging as critical regulators of immunocompetence. Among the 20 amino acids edited by gene codons, glutamine appears particularly important, contributing to bioenergetic as well as biosynthetic processes, while also helping to maintain redox balance. Glutamine is the most abundant and widely used amino acid in the human body. Glutamine is largely anaplerotic and relinquishes both of its amino groups to fuel the TCA cycle. It participates in the inter-organ nitrogen exchange through ammonia (NH3) transport between tissues and is critically involved in maintaining pH stability. Glutamine serves as a carbon and nitrogen donor for nucleotide biosynthesis and is a requirement for nicotinamide adenine dinucleotide phosphate (NADPH) generation. Thus, glutamine is an alternative fuel source, serves as a biosynthetic material, contributes to epigenetic and posttranslational modifications and determines the redox status, identifying this amino acid as a central regulator of immune cell fitness (88, 89).

### Glutamine to the Rescue: Maintaining Energy Production in Unfit Mitochondria

Glutamine, and its breakdown product glutamate, have both been identified as critical energy carriers in tumors, and the glutaminolytic pathway is considered a potential therapeutic target to suppress tumor growth (90). Both glucose and glutamine can provide carbons to feed the mitochondrial TCA chain and preference for one over the other may depend on local availability but may also affect the outcome of pathogenic immune responses. Glucose is primarily supplied by the liver, glutamine is synthesized by the muscle (91, 92), implicating these two organ systems in regulating protective and pro-inflammatory immunity. To make glutamine available for ATP generation, T cells utilize the Glutaminase isoenzymes (GLS1 and GLS2). Transcriptomic studies combined with metabolomic studies have defined GLS1 as a marker enzyme of fibroblast-like synoviocytes (FLS) isolated from RA patients (93). Notably, withdrawal of glutamine, but not of glucose, reduced RA-FLS proliferation, suggesting that the amino acid is critical in supporting pannus formation in the arthritic joint. Glucose may be a limiting factor, required to support multiple cell types in the inflamed synovial membrane, while glutamine may be freely accessible, excluding the proliferative FLS from glucose competition. Takahashi et al. have examined which factors can upregulate GLS1 in RA-FLS and found that both, IL-17 and platelet-derived growth factor acted as GLS1 inducers (93). The emerging model suggests that the T cell cytokine IL-17 directs synovial stromal cells to utilize glutamine instead of glucose, implicating metabolic regulation in the coordination of innate and adaptive immune responses in RA.

If the rheumatoid joint is a glutamine rich environment, then breakdown products derived from glutamine could potentially have pro-inflammatory functions. Through a series of enzymes, T cells convert glutamine into glutamate, which then is transformed into α-Ketoglutarate (α-KG) (94). The oncometabolite α-KG has versatility and has access to multiple cellular compartments, including the mitochondria, the cytosol, and the nucleus (95–97). α-KG can directly enter the TCA cycle, where it can be metabolized through oxidation and reduction. Conversion into succinate is a rate-limiting step in oxidative phosphorylation. Transformation into citrate feeds carbons into lipid metabolism. By functioning as a precursor for glutamine, α-KG also takes a center position in maintaining the redox balance of the cell. Given the enrichment in glutamate, discussions have focused on whether glutamatergic signaling has a role in the pathophysiology of RA (98). Measurements in collagen-induced arthritis (CIA) have shown a marked increase of glutamate in the synovial fluid. Glutamate functioned as an arthritogenic effector molecule by driving proliferation of synovial fibroblasts. Further support for a direct contribution of glutamate in the disease process came from blocking studies, applying Memantine, an N-methyl-D-aspartate (NMDA) ionotropic glutamate receptor antagonist. This receptor antagonist had anti-inflammatory efficacy by upregulating CD4^+^CD25^+^ regulatory T cells in the spleen (99). These data suggest that glutamate is not only a stimulatory neurotransmitter, but it also has immunoregulatory functions. To better understand how glutamate promotes disease, it would be important to know the cellular origin and the glutamate receptor expression profile of innate, adaptive, and stromal cells participating in synovitis.

A particularly appealing concept is the idea that the tolerance-breaking effect of glutamate is a consequence of its function as a neurotransmitter. Support for this concept has come from studies applying the non-competitive NMDA ionotropic glutamate receptors antagonist ketamine intrathecally (100). When injected intrathecally into animals with antigen-induced arthritis, arthritis severity is reduced, the density of inflammatory cells in the joints is lowered and joint destruction is halted (100). Therapeutic application would require a more precise understanding of which cells are signal-sending and which cells are signal-receiving.

### Pumping in Amino Acids: Glutamine Transporters as Disease-Promoting Molecules

Mechanistic studies have attempted to define upstream mediators that trigger the enhancement of glutaminolysis in T cells. During T cell activation, glutamine transporters and the components of the glutaminolysis machinery are upregulated through a MYC-dependent pathway (101). Compared to the resting state, activated T cells increase their glutamine uptake 5-10-fold. Several amino acid transporters facilitate glutamine uptake in T cells; e.g., the sodium-coupled neutral amino acid transporters of the SLC38 gene family (102). Upon T cell activation, transcription of SLC38A1 and SLC38A2 is activated in a coordinated fashion. Interestingly, SLC38A1 and SLC38A2 protein is stored in intracellular vesicles from where it is rapidly transferred to the cell surface (103). Early events after T cell receptor triggering, such as membrane-proximal signaling and induction of T cell activation markers are unaffected by the withdrawal of glutamine. Conversely, late processes, such as T cell clonal expansion and cytokine release are highly sensitive to the lack of glutamine, predicting that glutamine is partially important for pro-inflammatory effector functions of T cells.

Several transporters ensure sufficient glutamine uptake in T cells undergoing activation, including SLC1A5 (104). Naïve CD4^+^ T cells rely upon SLC1A5 to fulfill their need for glutamine. In line with the concept that T cell activation and differentiation require metabolic adaptation, SLC1A5 couples TCR and CD28 signals to ultimately activate the mTORC1 pathway and enable the cell to undergo clonal expansion (105). SLC1A5 expression seems to be a prerequisite for the lineage commitment of both Th1 and Th17 cells (105), placing it high on the list of potential upstream regulators of the pathogenic differentiation of short-lived effector T cells encountered in RA patients (31). No information is currently available how a specific tissue microenvironment affects metabolically regulated aspects of T cell differentiation. Considering that T cell pathology in RA occurs both in lymphoid tissues as well as the inflamed joint, it would be important to know whether differences exist for glutamine availability, to which degree glutamine substitutes as an energy carrier under conditions of glucose deprivation and how glutamine interferes with the pathogenic mTORC activation driving disease-relevant effector functions.

## Lipids as a Catalyst for Autoimmune Joint Inflammation

Like glucose and amino acids, lipids are an essential nutrient resource for protective and pathogenic immune cells. Lipids are a high-impact energy source, affecting multiple biological processes, including the production and the storage of energy (106), the assembly and functionality of cellular membranes (107), gene regulation of metabolic pathways (108) and post-translational modifications in intracellular signaling pathways (109). Intracellular lipid concentrations are regulated both by nutritional uptake and by ongoing biosynthesis. Fatty acid synthesis involves the creation of fatty acids from acetyl-CoA and NADPH through the action of enzymes called fatty acid synthases (FAS), through which simple lipids are bio-synthesized and sequentially elongated through addition of acetyl-CoA (110).

### Disordered Lipid Profiles in Inflammatory Disease

Numerous autoimmune diseases, including RA, psoriasis, and systemic lupus erythematosus, in which autoreactive CD4^+^ T cells participate in inflammatory tissue lesions share the accumulation of intracellular lipid droplets in such T cells (111–113). These autoimmune diseases are associated with elevation of serum triglycerides and cholesterol (113–115). Whether this dyslipidemia is causative for inflammation or a consequence of persistent inflammatory activity remains unresolved. Evidence for a disease-relevant role of lipids has come from reports that lowering blood lipid levels by diet or drug treatment may improve symptoms, including T cell-dependent autoantibody responses (116–118). Nevertheless, it remains speculative whether modification of lipid profiles could reestablish tissue tolerance and revert pathogenic immunity.

Lipids accumulating in the synovial fluid of RA patients have been analyzed through liquid chromatography mass spectrometry, and more than 70 different components from different lipid classes have been detected (119). Among the broad spectrum of lipids found in synovial fluid, maresin 1, lipoxin A4 and resolvin D5 were associated with RA and 5S,12S-6E,8Z,10E,14Z-dihydroxyeicosatetraenoic acid was identified as a marker of lipoxygenase activity. Nevertheless, lipidomic studies have not yielded any unexpected insights. In early studies more than 50 years ago, increased amounts of phospholipid, cholesterol, and neutral lipids were described, but synovial fluid lipid levels were not predictive of severity of synovitis, questioning a direct involvement of lipids in driving arthritis (120, 121).

The abnormal cholesterol profile in patients with preclinical RA and early RA is typical of metabolic syndrome: normal or mildly elevated total cholesterol, LDL cholesterol and triglycerides, associated with decreased HDL cholesterol levels (122, 123). The cholesterol biosynthesis pathway appears to be a key regulator of controlling CD4^+^ T cell commitment to inflammatory versus anti-inflammatory effector status. Inhibition of the cholesterol biosynthesis pathway produces a specific block in immune resolution, defined as a significant decrease of T cell IL-10 production (124). Thus, anti-inflammatory response in CD4^+^ T cells may be particularly dependent on sterol metabolism.

### Cytoplasmic Lipid Droplets As Immunoregulatory Organelles

Intracellular lipids, however, play a quintessential role in how immune cells differentiate into effector cells. Indeed, the activation of the T cell receptor is coordinated with up-regulation of genes involved in the biosynthesis of cholesterol and fatty acids (125). This anabolic program is regulated by specialized transcription factors, the sterol regulatory element-binding proteins (SREBP) (125). Without SREBP signaling, T effector cells could not blast and could not undergo clonal expansion (125). T cell activation is associated with massive cellular proliferation, imposing high biosynthetic demands, particularly when it comes to the generation of bio-membranes. Besides their essential role as biosynthetic precursor, lipids also serve a non-replaceable role a bioenergetic materials, specifically in the induction of T memory cells, which are known to dependent on mitochondrial fatty acid β-oxidation (126). Notably, T memory cells, unlike T effector cells, do not uptake extracellular palmitate (126), delineating a stringent association between T cell subtype and preferences for fatty acid substrates. How different T cell subpopulations acquire and mediate their taste for select lipids is not entirely clear. Different fatty acid transporters are expressed on the membrane of distinct T cell subsets. In the case of memory T cells, survival is impaired in the presence of the FAS inhibitor C75 (127), indicating that the fitness of T memory cells depends on *de novo* fatty acid biosynthesis.

**Figure 4:**
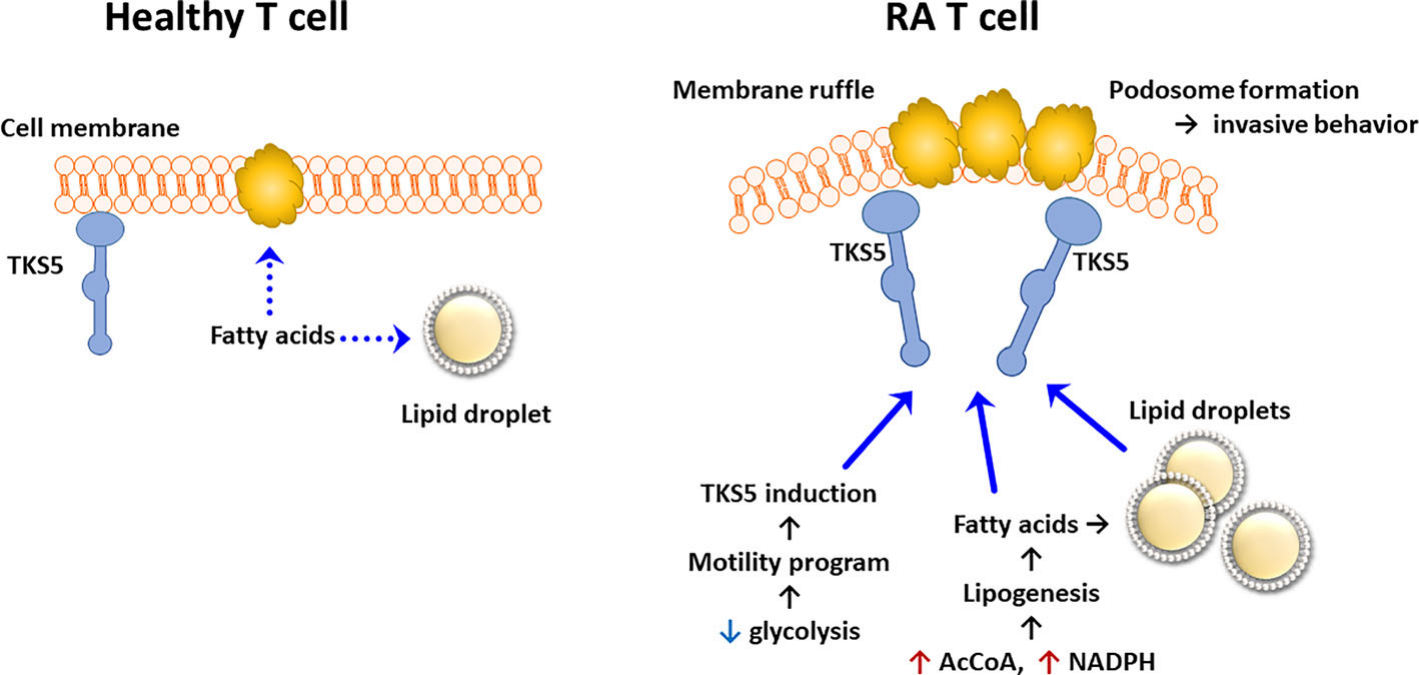
Pathogenic Lipogenesis in RA T cells. Metabolic rewiring in RA T cells, including the shunting of glucose to the pentose phosphate pathway, the export of mitochondrial citrate and the suppression of mitochondrial lipid oxidation, all favor lipogenesis over lipolysis. As a result, RA T cells deposit lipid droplets in the cytoplasm and are rich in biosynthetic precursors for membrane lipids. Lowered glycolytic activity upregulates a module of motility genes, including the scaffolding protein TKS5. As part of their pro-inflammatory phenotype, lipogenesis-biased TKS5^hi^ RA T cells spontaneously form actin- and cortactin-rich membrane ruffles, equipping them tissue invasion and mobility in extracellular matrix. AcCoA, acetyl-CoA.

Data that have accumulated over the last 5 years support the concept that lipid metabolism in RA T cells is fundamentally abnormal. Due to the mitochondrial defects (**Figure 3**), RA T cells lack the flexibility to use lipids as an energy carrier. Instead, RA T cells commit fatty acids to the lipogenesis program to support the biomass building program. Excess lipids that cannot be metabolized are stored as cytoplasmic lipid droplets (29, 128). Fatty acids that are not committed to the building of cellular offspring need to be safely packed and stored. Notably, tissue-residing T cells in rheumatoid synovitis accumulate cytoplasmic lipid droplets (128). Currently, there is no evidence that the lipogenesis>>lipolysis status of RA T cells is a primary defect, but rather appears to be a consequence of abnormal mitochondrial function and rerouting of glucose into the biosynthetic PPP. Reversal of the TCA cycle and the export of citrate out of the mitochondria leads to the accumulation of acetyl-CoA in the cytoplasm (129, 130). Fatty acid synthesis is further facilitated by the availability of NADPH, a cofactor required for lipogenesis and available due to the commitment to the PPP instead of glycolysis (131). Surplus in fatty acids has direct impact on the pathogenic effector functions of RA T cells, as fatty acids can be integrated into phospholipids, creating building blocks for cell membranes. Data on the composition of cellular and subcellular membranes in RA T cells are currently not available, but structural analysis has yielded insights on how these autoimmunity-prone cells adapt to their metabolic programming (132). Colocalization studies of the cytoskeletal marker F-actin and the membrane marker cortactin have demonstrated membrane extrusions indicative of rapid membrane turnover and structural flexibility (133, 134). Membrane ruffling occurs in cells loaded with cytoplasmic lipid droplets and translates into high mobility in 3-dimensional matrix and rapid transition from the blood to the tissue (128). Acetyl-rich RA T cells easily change their cellular shape, building a uropod known to promote cellular motion (29). Studies applying metabolic inhibitors have placed the redirection of glucose and lipids upstream of membrane ruffling and podosome formation (**Figure 4**) (128). Nevertheless, the question arises whether the loss of a circular cellular shape has metabolic implications by itself. Analysis of the content of the uropod in RA T cells has revealed the displacement of mitochondria into a perinuclear position (29). The mitochondrial rearrangement is rooted in the hyperacetylation of cytoskeletal proteins, specifically tubulin, which propels mitochondria away from the periphery close to the nuclear membrane. The proximity of ROS-producing mitochondria to the nucleus and to chromosomes placed near the nuclear membrane enables mitochondria to impose gene expression changes that by itself can mediate information from the metabolic hub of the cell to the “cockpit”. Essentially, metabolic rewiring may function as a feed forward mechanism, profoundly altering programs that oversee cellular behavior and adaptive immunity.

## Metabolic Networks as Therapeutic Targets

Immunomodulatory therapies currently applied in RA have metabolic “side effects” (**Table 1**). It is difficult to say whether the metabolic consequences induced by these therapeutics are primary or secondary. Obviously, effective control of systemic inflammation may leave a metabolic footprint, even if the pathways of intracellular metabolic control are ignored by the therapeutic. Some of these immunosuppressive medications may directly interfere with glycolysis, mitochondrial function, lipogenesis, and amino acid metabolism, but mechanistic understanding of such effects remains scarce. The gold standard of RA management, Methotrexate (MTX), is a potent antimetabolite drug that targets the processing of purine (155) and folic acid (156) by inhibiting dihydrofolate reductase. MTX, corticosteroids, sulfasalazine, leflunomide and cyclosporine A function as immunomodulators by inhibiting pro-inflammatory Th1 and Th2-dependet immunity (157). However, their metabolic impact is not always protective. Corticosteroids as well as nonsteroidal anti-inflammatory drugs have metabolic consequences that enhance cardiovascular risk (158).

**Table 1:** Metabolic effects of currently used therapies in rheumatoid arthritis.

Therapies for RA	Metabolic effects	References
Methotrexate	Accumulation of polyamines, inhibition of purine and pyrimidine synthesis, promotion of adenosine release	(135–138)
Anti-TNF	Decreased insulin serum levels, insulin resistance, increased HDL cholesterol serum levels, hypertriglyceridemia	(139–145)
Anti-CD20	Decreased succinate, taurine, lactate, pyruvate and aspartate in serum	(146)
Hydroxychloroquine	Decreased total cholesterol, decreased LDL, increased HDL and decreased triglycerides	(147–150)
Anti-IL-6	Altered body composition, increased lean mass and skeletal muscle mass, decreases oxidative stress in leucocytes	(151, 152)
Leflunomide	Decreased synthesis of pyrimidine, decreased uric acid	(153, 154)

Anti-TNF-α drugs have become a cornerstone in managing RA, particularly in patients resistant to traditional therapeutic approaches (159, 160). Anti-TNF-α treatment decreases glycolytic activity in RA synovium, likely through its potent anti-inflammatory effect (161). Blockade of IL-6 appears to have primarily a systemic are metabolic impact. Anti-IL-6 receptor treatment leads to weight gain and modified fat distribution and gain in muscle mass suggests that blocking IL-6 might be efficient in treating sarcopenia associated with RA (151). Anti-IL-6 receptor treatment has been reported to decreases oxidative stress in RA leucocytes (152). Inhibitors of the JAK-STAT pathway (e.g. Tofacitinib) display powerful anti-inflammatory effects and significantly decrease mitochondrial membrane potential, mitochondrial mass and ROS production in RA synovial fibroblasts (162). In an elegant study, McGarry et al. have provided evidence that tofacitinib directly modulates mitochondrial function, such as regulating key mitochondrial genes, increasing oxidative phosphorylation, enhancing ATP production while diminishing glycolytic flow and key glycolytic genes (162).

Capitalizing on the understanding of how fuel selection guides cellular behavior and on the concept that fuel determines function, multiple novel therapeutic targets emerge. Besides the central role of glucose itself (163), metabolic intermediates such as lactate (53) and succinate (70) appear to be critically important in regulating tissue inflammation. Much will be learned from therapeutic approaches in cancer therapy that rely on disrupting bioenergetic needs to the tumor and metabolic vulnerabilities in anti-tumor immune responses (164). Unselected approaches, such as shutting off glucose consumption, amino acid utilization or lipid import are likely going to fail, as all organ systems are dependent on energy supply from these energy carriers (165) and more sophisticated strategies are needed to manipulate cellular metabolism.

## Conclusion

Autoimmune diseases, such as RA, begin with T cells losing self-tolerance and providing help to auto-antibody producing B cells. The root cause is believed to be the recognition of autoantigen and the failure of mechanisms that sort out autoreactive T cells. Recent data have questioned this simplified model and have added metabolic signals as decisive determinants in T cell fate and behavior. Based on the concept that “fuel feeds function”, the metabolic networks of disease-inducing T cells have been defined and are being explored as liabilities that may be targeted to re-engineer “bad” T cells into “good” T cells. An additional layer of metabolic control is introduced by the metabolic cues provided by the tissue environment in which cells live, die, proliferate, and communicate. RA CD4^+^ T cells lose tolerance decades before joint inflammation begins, while living in lymphoid organs, such as lymph nodes and the bone marrow. Once such T cells differentiate into short-lived effector cells (SLEC) instead of long-lived memory cells, they function as tissue-infiltrating and pro-inflammatory effector cells.

**Figure 5:**
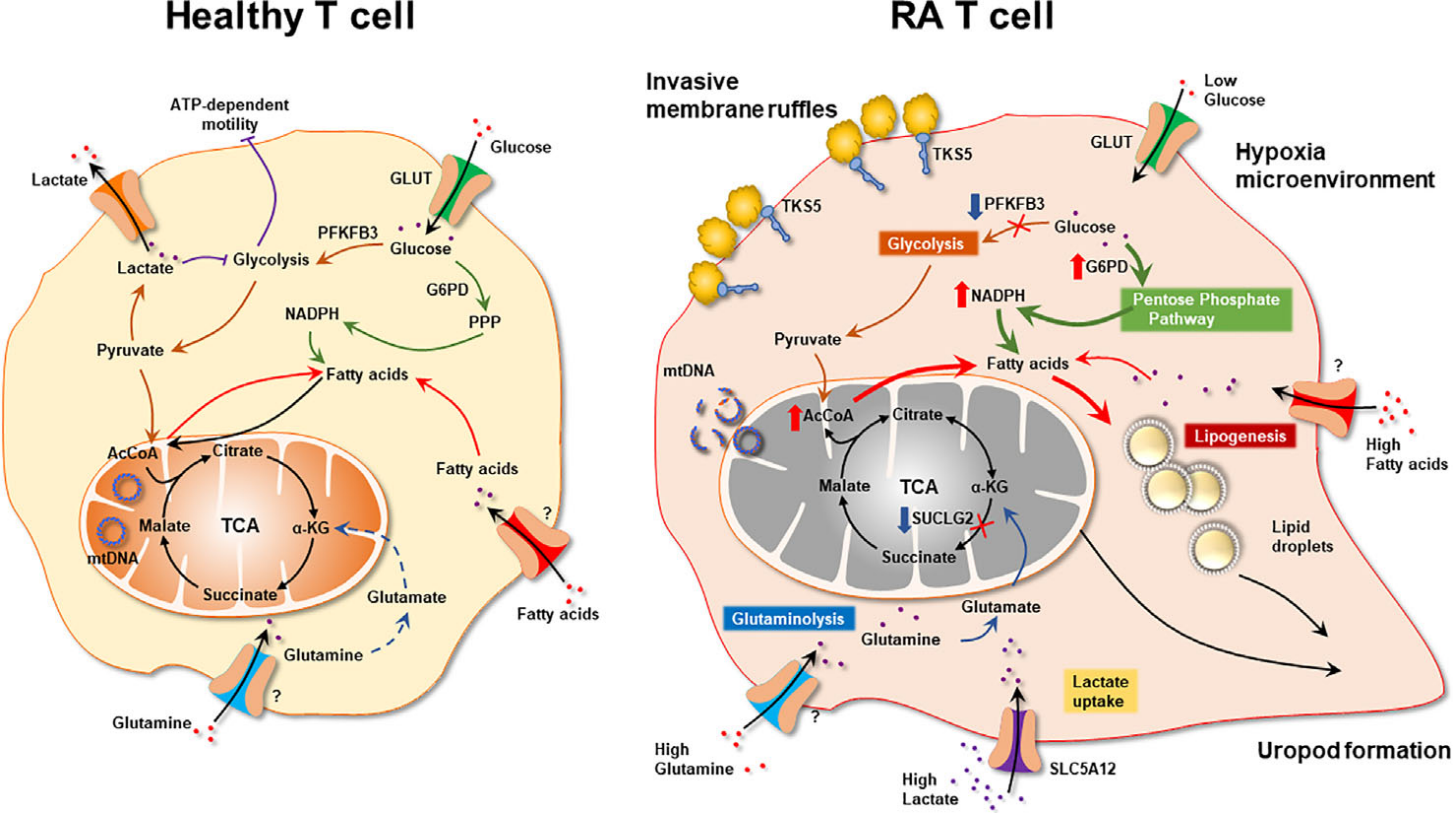
The Metabolic Signature of Autoreactive T cells in Rheumatoid Arthritis. Schematic diagram of metabolic pathways in health and RA T cells. Healthy T cell: Low-proliferating healthy T cells (naïve, memory) rely on mitochondrial energy production. When differentiating into highly replicating effector T cells, bioenergetic and biosynthetic needs are fulfilled by engaging glycolysis. RA T cells: Biased toward biomass generation, RA T cells shunt glucose into the pentose phosphate pathway and produce NADPH. Mitochondria with unrepaired mtDNA utilize relatively low amounts of oxygen and produce low concentrations of ROS. Mitochondrial succinate production is suppressed due to low activity of Succinate-CoA ligase and a-ketoglutarate is converted into citrate. Exported citrate supplies cytoplasmic acetyl-CoA pools and promotes lipogenesis. Surplus lipids sustain the generation of invasive membrane ruffles and of cellular uropods. With low ATP production, RA T cells require external energy sources, such as glutamine and lactate to meet basic bioenergetic needs. By diverting energy away from ATP production toward assembly of biosynthetic precursor molecules, RA T nourish the emergence and the survival of replicating effector T cells that are tissue-invasive and pro-inflammatory. α-KG, α-ketoglutarate; mtDNA, mitochondrial DNA.

**Figure 6:**
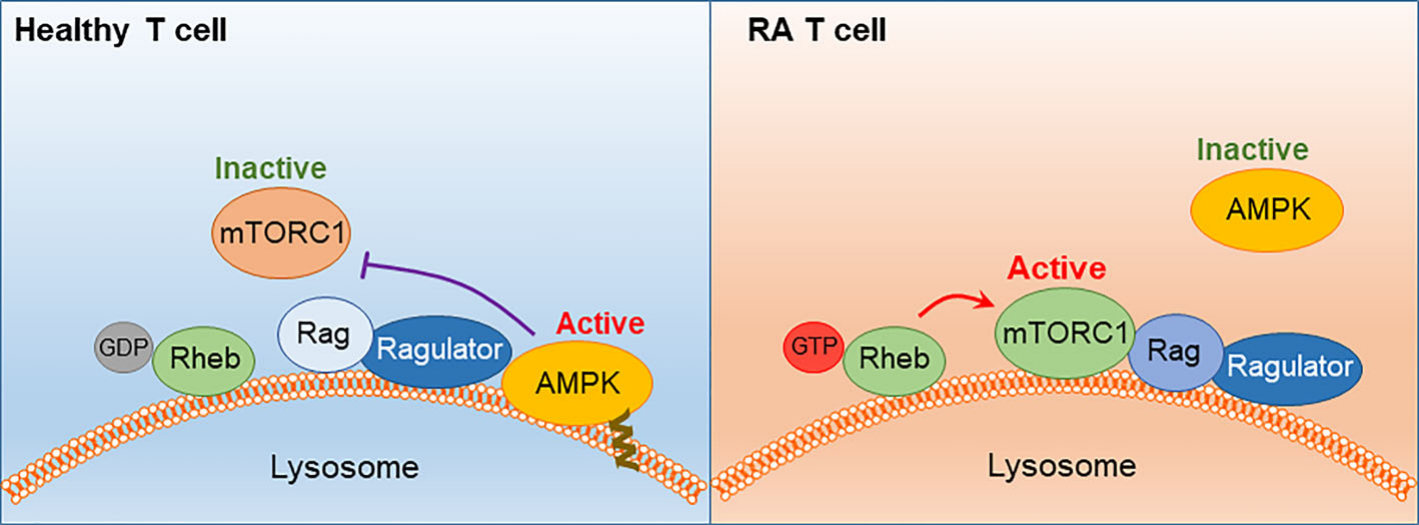
AMPK signaling failure and unopposed mTORC1 activation in RA T cells. In healthy T cells, AMPK senses the cell’s energy state by trafficking to the lysosomal surface, where it is anchored in the lysosomal membrane through a myristoylated tail. AMPK interacts with the Ragulator-Rag complex, resulting in dissociation and inactivation of mTORC1. In RA T cells, a defect in protein myristoylation leads to mistrafficking and cytosolic retention of AMPK. mTORC1 is retained at the lysosomal surface, where Rheb-GDP converts into Rheb-GTP and switches on the kinase activity of mTORC1. mTORC1 activation persists in RA T cells despite low ATP availability.

Here are the hallmarks of altered immunometabolic regulation in RA:

- RA T cells are characterized by abnormal glycolysis and inappropriate lipogenesis (**Figure 5**).
- The underlying defect is a malfunctioning mitochondrion. Lacking DNA repair of the mitochondrial genome leads to DNA instability and cytosolic leakage. Triggering of the inflammasome gives rise to T cell pyroptosis, a process that by itself functions as a strong instigator of tissue inflammation.
- Insufficient mtDNA damage repair undermines OxPhos and ATP generation. The mitochondrial TCA cycle is disrupted due to transcriptional repression of *SUCLG2*, impairing the conversion of α-KG into succinate. Unprocessed α-KG leads to reversal of the TCA cycle and results in the accumulation and the export of citrate.
- This cataploretic step promotes acetyl-CoA accumulation in the cytoplasm and favors acetylation of cytoplasmic proteins. Hyperacetylation of microtubules stiffens the cytoskeleton, fundamentally changing the cellular shape and the distribution of subcellular organelles. The most significant consequence of the hyperacetylate state is transfiguration of the cell away from a circular shape toward rear-front polarization and uropod formation. The resulting cell is hypermobile, swiftly transmigrating from the blood to the tissue.
- Besides the change in migratory behavior and invasiveness, RA T cells differentiate into pro-inflammatory cytokine producers, favoring IFN-γ and IL-17 as effector cytokines. The commitment to Th1 and Th17 differentiation results from the persistent activation of mTORC1, a consequence of the misplacement of the energy sensor AMPK. Lacking a myristoylation tail, AMPK in RA T cells fails to anchor on the lysosomal surface and thus forgoes the suppression of mTORC1 (**Figure 6**). Unopposed mTORC1 activation encourages RA T cells to proliferate and build daughter cells despite the low ATP state. The proliferation program of SLECs, however, is supported by the shunting of glucose from bioenergetic to biosynthetic utilization. Instead of breaking glucose down into ATP and pyruvate, RA T cells shift glucose into the PPP and generate NADPH plus biosynthetic precursors. Ongoing lipogenesis provides building blocks for membranes and organelles and the cells’ reductive state promotes fatty acid synthesis.
- Once transitioned into the inflamed joint, RA T cells encounter a hypoxic, lactate-rich, glucose- low environment. The chronic activation of stromal cells and invading immune cells depletes the tissue of glucose and acidifies the environment with lactate. Synovial T cells have been shown to uptake lactate, supplementing their energy supply but also signaling the cells to undergo arrest and persist in the tissue niche.
- Most metabolic abnormalities in RA T cells are present in the naïve population and sustained in tissue-residing memory T cells, placing metabolic dysregulation upstream of the joint. These data classify the metabolic malfunction, at least in part, as an “original sin”, leading to autoimmunity.

Based on the novel paradigm that metabolic programming determines the risk for inappropriate immune function, new therapeutic interventions can be developed. Ideally, a dietary approach can be designed to direct the flow of energy carriers and that of intracellular and extracellular metabolites. Alternatives include small molecules designed to finetune glycolysis, glutaminolysis and lipogenesis. Metabolic intervention may eventually be the most elegant way to manipulate the energy sensor mTORC1, which is critically involved in misleading T cells and turning them into multiplying and self-aggressive effector cells.

## Author Contributions

All authors contributed to concept development. JQ, BW, GB, SG, JG and CW wrote the manuscript. All authors contributed to the article and approved the submitted version.

## Funding

This work was supported by the National Institutes of Health (R01 AR042527, R01 HL117913, R01 AI108906, R01 HL142068, and P01 HL129941 to CW and R01 AI108891, R01 AG045779, U19 AI057266, and R01 AI129191 to JG. Merit Review Award I01 BX001669 from the United States (U.S.) Department of Veterans Affairs to JG. JQ and BW were supported by the Encrantz Family Discovery Fund.

## Conflict of Interest

The authors declare that the research was conducted in the absence of any commercial or financial relationships that could be construed as a potential conflict of interest.
